# Supplementary material for: Punctuated evolution and transitional hybrid network in an ancestral cell cycle of fungi
Source: eLife. 2016 May 10;5:e09492. doi: 10.7554/eLife.09492 (PMC4862756; doi:10.7554/eLife.09492)
Supplement: Supplementary file 1. — (A) List of eukaryotic genomes. We downloaded and analyzed the following annotated genomes using the 'best' filtered protein sets when available. We gratefully acknowledge the Broad Institute, the DOE Joint Genome Institute, Génolevures, PlantGDB, SaccharomycesGD, AshbyaGD, DictyBase, JCV Institute, Sanger Institute, TetrahymenaGD, PythiumGD, AmoebaDB, NannochloroposisGD, OrcAE, TriTryDB, GiardiaDB, TrichDB, CyanophoraDB, and CyanidioschizonDB for making their annotated genomes publicly available. We especially thank D. Armaleo, I. Grigoriev, T. Jeffries, J. Spatafora, S. Baker, J. Collier, and T. Mock for allowing us to use their unpublished data. (B) Plasmids. (C) Strains. All yeast strains were derived from W303 and constructed using standard methods. DOI: http://dx.doi.org/10.7554/eLife.09492.033 [file elife-09492-supp1.docx]

| Species genus | Genome source: | References: |
| --- | --- | --- |
| *Saccharomyces cerevisiae* | Sacch. Genome Database | (Goffeau et al., 1996) |
| *Saccharomyces mikatae* | Sacch. Genome Database | (Cliften et al., 2003; Kellis et al., 2003) |
| *Saccharomyces bayanus* | Sacch. Genome Database | (Cliften et al., 2003; Kellis et al., 2003) |
| *Candida glabrata* | Genolevures | (Dujon et al., 2004) |
| *Zygosaccharomyces rouxii* | Genolevures | (Génolevures Consortium et al., 2009) |
| *Kluyveromyces waltii* | Broad Institute | (Kellis et al., 2004) |
| *Kluyveromyces thermotolerans* | Genolevures | (Génolevures Consortium et al., 2009) |
| *Saccharomyces kluyveri* | Genolevures | (Génolevures Consortium et al., 2009) |
| *Ashbya gossypii* | Ashbya Genome Database | (Dietrich et al., 2004) |
| *Kluyveromyces lactis* | Genolevures | (Dujon et al., 2004) |
| *Wickerhamomyces anomalus* | Center for Biotech., Bielefeld | (Schneider et al., 2012) |
| *Candida parapsilosis* | Broad Institute | (Butler et al., 2009) |
| *Candida albicans* | Broad Institute | (Butler et al., 2009) |
| *Candida tropicalis* | Broad Institute | (Butler et al., 2009) |
| *Candida guilliermondii* | Broad Institute | (Butler et al., 2009) |
| *Debaryomyces hansenii* | Genolevures | (Dujon et al., 2004) |
| *Candida lusitaniae* | Broad Institute | (Butler et al., 2009) |
| *Yarrowia lipolytica* | Genolevures | (Dujon et al., 2004) |
| *Neurospora crassa* | Broad Institute | (Galagan et al., 2003) |
| *Podospora anserina* | Genoscope | (Espagne et al., 2008) |
| *Magnaporthe grisea* | Broad Institute | (Dean et al., 2005) |
| *Fusarium graminearum* | Broad Institute | (Ma et al., 2010) |
| *Cladonia grayi* | DOE Joint Genome Institute | D. Armaleo (Duke) |
| *Aspergillus nidulans* | Broad Institute | (Galagan et al., 2005) |
| *Cocciodioides immitis* | Broad Institute | (Sharpton et al., 2009) |
| *Saitoella complicata* | DOE Joint Genome Institute | T. Jeffries (Wisconsin) |
| *Pneumocystis jirovecii* | DOE Joint Genome Institute | (Cissé et al., 2012) |
| *Taphrina deformans* | DOE Joint Genome Institute | (Cissé et al., 2013) |
| *Schizosaccharomyces pombe* | Broad Institute | (Rhind et al., 2011) |
| *Schizosaccharomcyes octosporus* | Broad Institute | (Rhind et al., 2011) |
| *Schizosaccharomyces japonicus* | Broad Institute | (Rhind et al., 2011) |
| *Coprinopsis cinerea* | Broad Institute | (Stajich et al., 2010) |
| *Laccaria bicolor* | DOE Joint Genome Institute | (Martin et al., 2008) |
| *Schizophyllum commune* | DOE Joint Genome Institute | (Ohm et al., 2010) |
| *Phanerochaete chrysosporium* | DOE Joint Genome Institute | (Martinez et al., 2004) |
| *Cryptococcus neoformans* | Broad Institute | (Loftus et al., 2005a) |
| *Ustilago maydis* | Broad Institute | (Kämper et al., 2006) |
| *Puccinia graminis* | Broad Institute | (Duplessis et al., 2011) |
| *Mortierella alpina* | Y.Q. Chen (Wake Forest) | (Wang et al., 2011) |
| *Mortierella verticillata* | Broad Institute | OriginsMultiCellularity |
| *Rhizophagus irregularis* | DOE Joint Genome Institute | (Tisserant et al., 2013) |
| *Umbelopsis ramanniana* | DOE Joint Genome Institute | J. Spatafora  (Oregon State) |
| *Lichtheimia hyalospora* | DOE Joint Genome Institute | J. Spatafora  (Oregon State) |
| *Mucor circinelloides* | Broad Institute | Mucorales database |
| *Phycomyces blakesleeanus* | DOE Joint Genome Institute | (2013) |
| *Rhizopus oryzae* | Broad Institute | (Ma et al., 2009) |
| *Coemansia reversa* | DOE Joint Genome Institute | I. Grigoriev (LBL) |
| *Conidiobolus coronatus* | DOE Joint Genome Institute | J. Spatafora  (Oregon State) |
| *Allomyces macrogynus* | Broad Institute | OriginsMultiCellularity |
| *Catenaria anguillulae* | DOE Joint Genome Institute | J. Spatafora  (Oregon State) |
| *Gonapodya prolifera* | DOE Joint Genome Institute | J. Spatafora  (Oregon State) |
| *Piromyces sp E2* | DOE Joint Genome Institute | S. Baker (PNL) |
| *Batrachochytrium dendrobatidis* | Broad Institute | OriginsMultiCellularity |
| *Spizellomyces punctatus* | Broad Institute | OriginsMultiCellularity |
| *Rozella allomycis* | DOE Joint Genome Institute | (James et al., 2013) |
| *Encephalitozoon cuniculi* | Broad Institute | (Katinka et al., 2001) |
| *Nosema ceranae* | Broad Institute | (Cornman et al., 2009) |
| *Edhazardia aedis* | Broad Institute | MicrosporidiaComp. |
| *Vittaforma corneae* | Broad Institute | MicrosporidiaComp. |
| *Nematocida parisii* | Broad Institute | (Cuomo et al., 2012) |
| *Fonticula alba* | Broad Institute | OriginsMultiCellularity |
| *Thecamonas trahens* | Broad Institute | OriginsMultiCellularity |
| *Sphaeroforma arctica* | Broad Institute | OriginsMultiCellularity |
| *Capsaspora owczarzaki* | Broad Institute | (Suga et al., 2013) |
| *Monosiga brevicollis* | DOE Joint Genome Institute | (King et al., 2008) |
| *Salpingoeca rosetta* | Broad Institute | (Fairclough et al., 2013) |
| *Trichoplax adhaerens* | DOE Joint Genome Institute | (Srivastava et al., 2008) |
| *Amphemidon queenslandica* | Ensembl | (Srivastava et al., 2010) |
| *Nemostella vectensis* | DOE Joint Genome Institute | (Putnam et al., 2007) |
| *Caenorhabditis elegans* | Ensembl | (C. elegans Sequencing Consortium, 1998) |
| *Drosophila melanogaster* | Ensembl | (Adams et al., 2000) |
| *Lottia gigantea* | DOE Joint Genome Institute | (Simakov et al., 2013) |
| *Strongylocentrotus purpuratus* | SpBase | (Sodergren et al., 2006) |
| *Ciona intestinalis* | DOE Joint Genome Institute | (Dehal et al., 2002) |
| *Branchiostoma floridae* | DOE Joint Genome Institute | (Putnam et al., 2008) |
| *Danio rerio* | Ensembl | (Howe et al., 2013) |
| *Gallus gallus* | Ensembl | (Hillier et al., 2004) |
| *Homo sapiens* | Ensembl | (Lander et al., 2001; Venter et al., 2001) |
| *Dictyostelium discoideum* | DictyBase | (Eichinger et al., 2005) |
| *Dictyostelium purpureum* | DOE Joint Genome Institute | (Sucgang et al., 2011) |
| *Dictyostelium fasciculatum* | DictyBase | (Heidel et al., 2011) |
| *Polysphondylium pallidum* | DictyBase | (Heidel et al., 2011) |
| *Entamoeba histolytica* | AmoebaDB | (Loftus et al., 2005b) |
| *Entamoeba nuttalli* | AmoebaDB |  |
| *Symbiodinium minutum* | OIST | (Shoguchi et al., 2013) |
| *Plasmodium falciparum* | PlasmoDB | (Gardner et al., 2002) |
| *Cryptosporidium muris* | CryptoDB | (Puiu et al., 2004) |
| *Toxoplasma gondii* | ToxoDB | (Gajria et al., 2008) |
| *Tetrahymena thermophila* | TetrahymenaGD | (Eisen et al., 2006) |
| *Guillardia theta* | DOE Joint Genome Institute | (Curtis et al., 2012) |
| *Emiliania huxleyi* | DOE Joint Genome Institute | (Read et al., 2013) |
| *Aurantiochytrium limacinum* | DOE Joint Genome Institute | J. Collier (Stony Brook) |
| *Schizochytrium aggregatum* | DOE Joint Genome Institute | J. Collier (Stony Brook) |
| *Phytophthora infestans* | Broad Institute | (Haas et al., 2009) |
| *Pythium ultimatum* | PythiumGD | (Lévesque et al., 2010) |
| *Hyaloperonospora parasitica* | Broad Institute | SaprolegniaProject |
| *Saprolegnia parasitica* | Broad Institute | SaprolegniaProject |
| *Fragilariopsis cylindrus* | DOE Joint Genome Institute | T. Mock (U.East Anglia) |
| *Phaeodactylum tricornutum* | DOE Joint Genome Institute | (Bowler et al., 2008) |
| *Thalassiosira pseudonana* | DOE Joint Genome Institute | (Armbrust et al., 2004) |
| *Nannochloropsis gaditana* | NannochGD | (Radakovits et al., 2012) |
| *Ectocarpus siliculosus* | OrcAE | (Cock et al., 2010) |
| *Aureococcus anophaefferens* | DOE Joint Genome Institute | (Gobler et al., 2011) |
| *Bigelowiella natans* | DOE Joint Genome Institute | (Curtis et al., 2012) |
| *Reticulomyxa filosa* | Genbank | (Glöckner et al., 2013) |
| *Giardia intestinalis* | GiardiaDB | (Franzén et al., 2009) |
| *Trichomonas vaginalis* | TrichDB | (Carlton et al., 2007) |
| *Naegleria gruberi* | DOE Joint Genome Institute | (Fritz-Laylin et al., 2010) |
| *Trypanosoma brucei* | TriTrypDB | (Berriman et al., 2005) |
| *Leishmania major* | Sanger Institute | (Ivens et al., 2005) |
| *Leishmania donovani* | TriTrypDB | (Downing et al., 2011) |
| *Cyanophora paradoxa* | CyanophoraGP | (Price et al., 2012) |
| *Porphyridium cruentum* | CyanophoraGP | (Bhattacharya et al., 2013) |
| *Cyanidioschyzon merolae* | CyanophoraGP | (Matsuzaki et al., 2004) |
| *Ostreococcus tauri* | DOE Joint Genome Institute | (Derelle et al., 2006) |
| *Ostreococcus lucimarinus* | DOE Joint Genome Institute | (Palenik et al., 2007) |
| *Micromonas pusilla* | DOE Joint Genome Institute | (Worden et al., 2009) |
| *Coccomyxa subellipsoidea* | DOE Joint Genome Institute | (Blanc et al., 2012) |
| *Volvox carteri* | PlantGDB | (Prochnik et al., 2010) |
| *Chlamydomonas reinhardtii* | PlantGDB | (Merchant et al., 2007) |
| *Physcomitrella patens* | PlantGDB | (Rensing et al., 2008) |
| *Selaginella moellendorffii* | PlantGDB | (Banks et al., 2011) |
| *Brachypodium distachyon* | PlantGDB | (International Brachypodium Initiative, 2010) |
| *Oryza sativa* | PlantGDB | (Goff et al., 2002) |
| *Arabidopsis thaliana* | PlantGDB | (Arabidopsis, 2000) |

## Supplementary File 1A: List of eukaryotic genomes. We downloaded and analyzed the following annotated genomes using the "best" filtered protein sets when available. We gratefully acknowledge the Broad Institute, the DOE Joint Genome Institute, Génolevures, PlantGDB, SaccharomycesGD, AshbyaGD, DictyBase, JCV Institute, Sanger Institute, TetrahymenaGD, PythiumGD, AmoebaDB, NannochloroposisGD, OrcAE, TriTryDB, GiardiaDB, TrichDB, CyanophoraDB, and CyanidioschizonDB for making their annotated genomes publicly available. We especially thank D. Armaleo, I. Grigoriev, T. Jeffries, J. Spatafora, S. Baker, J. Collier, and T. Mock for allowing us to use their unpublished data.

| Plasmid | Features | Source |
| --- | --- | --- |
| pLB02-0mer | *CLN2∆SCBpr-GFP-CLN2PEST* | (Bai et al., 2010) |
| pLB02-Cln2 | *CLN2pr-GFP-CLN2PEST* | This study |
| pLB02-E2F | *CLN2/E2Fpr-GFP-CLN2PEST* | This study |

**Supplementary File 1B:** **Plasmids.**

| Strain | Genotype | Source |
| --- | --- | --- |
| JE103 | MAT**a** *ADE2* | This study |
| JTY2 | MAT**a** *ADE2* *CLN2pr-GFP-CLN2PEST::*Ca*URA3::CLN2* | This study |
| JTY3 | MAT**a** *ADE2* *CLN2/E2Fpr-GFP-CLN2PEST::*Ca*URA3::CLN2* | This study |
| JTY4 | MAT**a** *ADE2* *CLN2∆SCBpr-GFP-CLN2PEST::*Ca*URA3::CLN2* | This study |
| 2798-1B | MATα *ADE2* *swi4::LEU2 mbp1::URA3*  *trp1::TRP1-MET3pr-CLN2 CLN2pr-GFP-CLN2PEST -His3::CLN2* | Fred Cross |
| JS272-9d | MATα *ADE2 swi4::LEU2 trp1::TRP1-MET3pr-CLN2 CLN2/E2Fpr-GFP-CLN2PEST::*Ca*URA3::CLN2* | This study |
| JS272-14a | MAT**a** *ADE2 mbp1::URA3 trp1::TRP1-MET3pr-CLN2 CLN2/E2Fpr-GFP-CLN2PEST::*Ca*URA3::CLN2* | This study |

**Supplementary File 1C: Strains.** All yeast strains were derived from W303 and constructed using standard methods.

Adams, M.D., Celniker, S.E., Holt, R.A., Evans, C.A., Gocayne, J.D., Amanatides, P.G., Scherer, S.E., Li, P.W., Hoskins, R.A., and Galle, R.F. (2000). The genome sequence of Drosophila melanogaster. Science *287*, 2185–2195.

Arabidopsis, G.I. (2000). Analysis of the genome sequence of the flowering plant Arabidopsis thaliana. Nature *408*, 796.

Armbrust, E.V., Berges, J.A., Bowler, C., Green, B.R., Martinez, D., Putnam, N.H., Zhou, S., Allen, A.E., Apt, K.E., and Bechner, M. (2004). The genome of the diatom Thalassiosira pseudonana: ecology, evolution, and metabolism. Science *306*, 79–86.

Bai, L., Charvin, G., Siggia, E.D., and Cross, F.R. (2010). Nucleosome-Depleted Regions in Cell-Cycle-Regulated Promoters Ensure Reliable Gene Expression in Every Cell Cycle. Dev Cell *18*, 544–555.

Banks, J.A., Nishiyama, T., Hasebe, M., Bowman, J.L., Gribskov, M., dePamphilis, C., Albert, V.A., Aono, N., Aoyama, T., Ambrose, B.A., et al. (2011). The Selaginella genome identifies genetic changes associated with the evolution of vascular plants. Science *332*, 960–963.

Berriman, M., Ghedin, E., Hertz-Fowler, C., Blandin, G., Renauld, H., Bartholomeu, D.C., Lennard, N.J., Caler, E., Hamlin, N.E., and Haas, B. (2005). The genome of the African trypanosome Trypanosoma brucei. Science *309*, 416–422.

Bhattacharya, D., Price, D.C., Chan, C.X., Qiu, H., Rose, N., Ball, S., Weber, A.P., Arias, M.C., Henrissat, B., and Coutinho, P.M. (2013). Genome of the red alga Porphyridium purpureum. Nature Communications *4*.

Blanc, G., Agarkova, I., Grimwood, J., Kuo, A., Brueggeman, A., Dunigan, D.D., Gurnon, J., Ladunga, I., Lindquist, E., and Lucas, S. (2012). The genome of the polar eukaryotic microalga Coccomyxa subellipsoidea reveals traits of cold adaptation. Genome Biol *13*, R39.

Bowler, C., Allen, A.E., Badger, J.H., Grimwood, J., Jabbari, K., Kuo, A., Maheswari, U., Martens, C., Maumus, F., and Otillar, R.P. (2008). The Phaeodactylum genome reveals the evolutionary history of diatom genomes. Nature *456*, 239–244.

Butler, G., Rasmussen, M., Lin, M., Santos, M., Sakthikumar, S., Munro, C., Rheinbay, E., Grabherr, M., Forche, A., Reedy, J., et al. (2009). Evolution of pathogenicity and sexual reproduction in eight Candida genomes. Nature.

C. elegans Sequencing Consortium (1998). Genome sequence of the nematode C. elegans: a platform for investigating biology. Science *282*, 2012–2018.

Carlton, J.M., Hirt, R.P., Silva, J.C., Delcher, A.L., Schatz, M., Zhao, Q., Wortman, J.R., Bidwell, S.L., Alsmark, U.C.M., and Besteiro, S. (2007). Draft genome sequence of the sexually transmitted pathogen Trichomonas vaginalis. Science *315*, 207–212.

Cissé, O.H., Almeida, J.M.G.C.F., Fonseca, Á., Kumar, A.A., Salojärvi, J., Overmyer, K., Hauser, P.M., and Pagni, M. (2013). Genome sequencing of the plant pathogen Taphrina deformans, the causal agent of peach leaf curl. MBio *4*, e00055–13.

Cissé, O.H., Pagni, M., and Hauser, P.M. (2012). De novo assembly of the Pneumocystis jirovecii genome from a single bronchoalveolar lavage fluid specimen from a patient. MBio *4*, e00428–12.

Cliften, P., Sudarsanam, P., Desikan, A., Fulton, L., Fulton, B., Majors, J., Waterston, R., Cohen, B.A., and Johnston, M. (2003). Finding functional features in Saccharomyces genomes by phylogenetic footprinting. Science *301*, 71–76.

Cock, J.M., Sterck, L., Rouzé, P., Scornet, D., Allen, A.E., Amoutzias, G., Anthouard, V., Artiguenave, F., Aury, J.-M., and Badger, J.H. (2010). The Ectocarpus genome and the independent evolution of multicellularity in brown algae. Nature *465*, 617–621.

Cornman, R.S., Chen, Y.P., Schatz, M.C., Street, C., Zhao, Y., Desany, B., Egholm, M., Hutchison, S., Pettis, J.S., and Lipkin, W.I. (2009). Genomic analyses of the microsporidian Nosema ceranae, an emergent pathogen of honey bees. PLoS Pathog *5*, e1000466.

Cuomo, C.A., Desjardins, C.A., Bakowski, M.A., Goldberg, J., Ma, A.T., Becnel, J.J., Didier, E.S., Fan, L., Heiman, D.I., Levin, J.Z., et al. (2012). Microsporidian genome analysis reveals evolutionary strategies for obligate intracellular growth. Genome Res *22*, 2478–2488.

Curtis, B.A., Tanifuji, G., Burki, F., Gruber, A., Irimia, M., Maruyama, S., Arias, M.C., Ball, S.G., Gile, G.H., and Hirakawa, Y. (2012). Algal genomes reveal evolutionary mosaicism and the fate of nucleomorphs. Nature *492*, 59–65.

Dean, R.A., Talbot, N.J., Ebbole, D.J., Farman, M.L., Mitchell, T.K., Orbach, M.J., Thon, M., Kulkarni, R., Xu, J.-R., and Pan, H. (2005). The genome sequence of the rice blast fungus Magnaporthe grisea. Nature *434*, 980–986.

Dehal, P., Satou, Y., Campbell, R.K., Chapman, J., Degnan, B., De Tomaso, A., Davidson, B., Di Gregorio, A., Gelpke, M., and Goodstein, D.M. (2002). The draft genome of Ciona intestinalis: insights into chordate and vertebrate origins. Science *298*, 2157–2167.

Derelle, E., Ferraz, C., Rombauts, S., Rouzé, P., Worden, A.Z., Robbens, S., Partensky, F., Degroeve, S., Echeynié, S., Cooke, R., et al. (2006). Genome analysis of the smallest free-living eukaryote Ostreococcus tauri unveils many unique features. *103*, 11647–11652.

Dietrich, F.S., Voegeli, S., Brachat, S., Lerch, A., Gates, K., Steiner, S., Mohr, C., Pöhlmann, R., Luedi, P., and Choi, S. (2004). The Ashbya gossypii genome as a tool for mapping the ancient Saccharomyces cerevisiae genome. Science *304*, 304–307.

Downing, T., Imamura, H., Decuypere, S., Clark, T.G., Coombs, G.H., Cotton, J.A., Hilley, J.D., de Doncker, S., Maes, I., and Mottram, J.C. (2011). Whole genome sequencing of multiple Leishmania donovani clinical isolates provides insights into population structure and mechanisms of drug resistance. Genome Res *21*, 2143–2156.

Dujon, B., Sherman, D., Fischer, G., Durrens, P., Casaregola, S., Lafontaine, I., De Montigny, J., Marck, C., Neuvéglise, C., Talla, E., et al. (2004). Genome evolution in yeasts. Nature *430*, 35–44.

Duplessis, S., Cuomo, C.A., Lin, Y.-C., Aerts, A., Tisserant, E., Veneault-Fourrey, C., Joly, D.L., Hacquard, S., Amselem, J., Cantarel, B.L., et al. (2011). Obligate biotrophy features unraveled by the genomic analysis of rust fungi. Proc Natl Acad Sci USA *108*, 9166–9171.

Eichinger, L., Pachebat, J.A., Glöckner, G., Rajandream, M.-A., Sucgang, R., Berriman, M., Song, J., Olsen, R., Szafranski, K., Xu, Q., et al. (2005). The genome of the social amoeba Dictyostelium discoideum. Nature *435*, 43–57.

Eisen, J.A., Coyne, R.S., Wu, M., Wu, D., Thiagarajan, M., Wortman, J.R., Badger, J.H., Ren, Q., Amedeo, P., and Jones, K.M. (2006). Macronuclear genome sequence of the ciliate Tetrahymena thermophila, a model eukaryote. PLoS Biol *4*, e286.

Espagne, E., Lespinet, O., Malagnac, F., Da Silva, C., Jaillon, O., Porcel, B.M., Couloux, A., Aury, J.-M., Ségurens, B., and Poulain, J. (2008). The genome sequence of the model ascomycete fungus Podospora anserina. Genome Biol *9*, R77.

Fairclough, S.R., Chen, Z., Kramer, E., Zeng, Q., Young, S., Robertson, H.M., Begovic, E., Richter, D.J., Russ, C., and Westbrook, M.J. (2013). Premetazoan genome evolution and the regulation of cell differentiation in the choanoflagellate Salpingoeca rosetta. Genome Biol *14*, 1–15.

Franzén, O., Jerlström-Hultqvist, J., Castro, E., Sherwood, E., Ankarklev, J., Reiner, D.S., Palm, D., Andersson, J.O., Andersson, B., and Svärd, S.G. (2009). Draft genome sequencing of giardia intestinalis assemblage B isolate GS: is human giardiasis caused by two different species? PLoS Pathog *5*, e1000560.

Fritz-Laylin, L.K., Prochnik, S.E., Ginger, M.L., Dacks, J.B., Carpenter, M.L., Field, M.C., Kuo, A., Paredez, A., Chapman, J., Pham, J., et al. (2010). The genome of Naegleria gruberi illuminates early eukaryotic versatility. Cell *140*, 631–642.

Gajria, B., Bahl, A., Brestelli, J., Dommer, J., Fischer, S., Gao, X., Heiges, M., Iodice, J., Kissinger, J.C., Mackey, A.J., et al. (2008). ToxoDB: an integrated Toxoplasma gondii database resource. Nucleic Acids Res *36*, D553–D556.

Galagan, J.E., Calvo, S.E., Borkovich, K.A., Selker, E.U., Read, N.D., Jaffe, D., FitzHugh, W., Ma, L.-J., Smirnov, S., and Purcell, S. (2003). The genome sequence of the filamentous fungus Neurospora crassa. Nature *422*, 859–868.

Galagan, J.E., Calvo, S.E., Cuomo, C., Ma, L.-J., Wortman, J.R., Batzoglou, S., and Su-In Lee, M.B.S. (2005). Sequencing of Aspergillus nidulans and comparative analysis with A. fumigatus and A. oryzae. Nature *438*, 1105–1115.

Gardner, M.J., Hall, N., Fung, E., White, O., and Berriman, M. (2002). Genome sequence of the human malaria parasite Plasmodium falciparum. Nature.

Génolevures Consortium, Souciet, J.-L., Dujon, B., Gaillardin, C., Johnston, M., Baret, P.V., Cliften, P., Sherman, D.J., Weissenbach, J., Westhof, E., et al. (2009). Comparative genomics of protoploid Saccharomycetaceae. Genome Res *19*, 1696–1709.

Glöckner, G., Hülsmann, N., Schleicher, M., and Noegel, A.A. (2013). The Genome of the Foraminiferan Reticulomyxa filosa. Current Biology.

Gobler, C.J., Berry, D.L., Dyhrman, S.T., Wilhelm, S.W., Salamov, A., Lobanov, A.V., Zhang, Y., Collier, J.L., Wurch, L.L., Kustka, A.B., et al. (2011). Niche of harmful alga Aureococcus anophagefferens revealed through ecogenomics. Proc Natl Acad Sci USA *108*, 4352–4357.

Goff, S.A., Ricke, D., Lan, T.-H., Presting, G., Wang, R., Dunn, M., Glazebrook, J., Sessions, A., Oeller, P., and Varma, H. (2002). A draft sequence of the rice genome (Oryza sativa L. ssp. japonica). Science *296*, 92–100.

Goffeau, A., Barrell, B.G., Bussey, H., Davis, R.W., Dujon, B., Feldmann, H., Galibert, F., Hoheisel, J.D., Jacq, C., and Johnston, M. (1996). Life with 6000 genes. Science *274*, 546–567.

Haas, B.J., Kamoun, S., Zody, M.C., Jiang, R.H., Handsaker, R.E., Cano, L.M., Grabherr, M., Kodira, C.D., Raffaele, S., and Torto-Alalibo, T. (2009). Genome sequence and analysis of the Irish potato famine pathogen Phytophthora infestans. Nature *461*, 393–398.

Heidel, A.J., Lawal, H.M., Felder, M., Schilde, C., Helps, N.R., Tunggal, B., Rivero, F., John, U., Schleicher, M., Eichinger, L., et al. (2011). Phylogeny-wide analysis of social amoeba genomes highlights ancient origins for complex intercellular communication. Genome Res *21*, 1882–1891.

Hillier, L.W., Miller, W., Birney, E., Warren, W., Hardison, R.C., Ponting, C.P., Bork, P., Burt, D.W., Groenen, M.A., and Delany, M.E. (2004). Sequence and comparative analysis of the chicken genome provide unique perspectives on vertebrate evolution. Nature *432*, 695–716.

Howe, K., Clark, M.D., Torroja, C.F., Torrance, J., Berthelot, C., Muffato, M., Collins, J.E., Humphray, S., McLaren, K., and Matthews, L. (2013). The zebrafish reference genome sequence and its relationship to the human genome. Nature.

International Brachypodium Initiative (2010). Genome sequencing and analysis of the model grass Brachypodium distachyon. Nature *463*, 763–768.

Ivens, A.C., Peacock, C.S., Worthey, E.A., Murphy, L., Aggarwal, G., Berriman, M., Sisk, E., Rajandream, M.-A., Adlem, E., and Aert, R. (2005). The genome of the kinetoplastid parasite, Leishmania major. Science *309*, 436–442.

James, T.Y., Pelin, A., Bonen, L., Ahrendt, S., Sain, D., Corradi, N., and Stajich, J.E. (2013). Shared Signatures of Parasitism and Phylogenomics Unite Cryptomycota and Microsporidia. Current Biology *23*, 1548–1553.

Katinka, M.D., Duprat, S., Cornillot, E., Méténier, G., Thomarat, F., Prensier, G., Barbe, V., Peyretaillade, E., Brottier, P., Wincker, P., et al. (2001). Genome sequence and gene compaction of the eukaryote parasite Encephalitozoon cuniculi. Nature *414*, 450–453.

Kämper, J., Kahmann, R., Bölker, M., Ma, L.-J., Brefort, T., Saville, B.J., Banuett, F., Kronstad, J.W., Gold, S.E., and Müller, O. (2006). Insights from the genome of the biotrophic fungal plant pathogen Ustilago maydis. Nature *444*, 97–101.

Kellis, M., Birren, B.W., and Lander, E.S. (2004). Proof and evolutionary analysis of ancient genome duplication in the yeast Saccharomyces cerevisiae. Nature *428*, 617–624.

Kellis, M., Patterson, N., Endrizzi, M., Birren, B., and Lander, E.S. (2003). Sequencing and comparison of yeast species to identify genes and regulatory elements. Nature *423*, 241–254.

King, N., Westbrook, M.J., Young, S.L., Kuo, A., Abedin, M., Chapman, J., Fairclough, S., Hellsten, U., Isogai, Y., Letunic, I., et al. (2008). The genome of the choanoflagellate Monosiga brevicollis and the origin of metazoans. Nature *451*, 783–788.

Lander, E.S., Linton, L.M., Birren, B., Nusbaum, C., Zody, M.C., Baldwin, J., Devon, K., Dewar, K., Doyle, M., and FitzHugh, W. (2001). Initial sequencing and analysis of the human genome. Nature *409*, 860–921.

Lévesque, C.A., Brouwer, H., Cano, L., Hamilton, J.P., Holt, C., Huitema, E., Raffaele, S., Robideau, G.P., Thines, M., and Win, J. (2010). Genome sequence of the necrotrophic plant pathogen Pythium ultimum reveals original pathogenicity mechanisms and effector repertoire. Genome Biol *11*, R73.

Loftus, B.J., Fung, E., Roncaglia, P., Rowley, D., Amedeo, P., Bruno, D., Vamathevan, J., Miranda, M., Anderson, I.J., and Fraser, J.A. (2005a). The genome of the basidiomycetous yeast and human pathogen Cryptococcus neoformans. Science *307*, 1321–1324.

Loftus, B., Anderson, I., Davies, R., Alsmark, U.C.M., Samuelson, J., Amedeo, P., Roncaglia, P., Berriman, M., Hirt, R.P., and Mann, B.J. (2005b). The genome of the protist parasite Entamoeba histolytica. Nature *433*, 865–868.

Ma, L.-J., Ibrahim, A.S., Skory, C., Grabherr, M.G., Burger, G., Butler, M., Elias, M., Idnurm, A., Lang, B.F., Sone, T., et al. (2009). Genomic analysis of the basal lineage fungus Rhizopus oryzae reveals a whole-genome duplication. PLoS Genet *5*, e1000549.

Ma, L.-J., van der Does, H.C., Borkovich, K.A., Coleman, J.J., Daboussi, M.-J., Di Pietro, A., Dufresne, M., Freitag, M., Grabherr, M., Henrissat, B., et al. (2010). Comparative genomics reveals mobile pathogenicity chromosomes in Fusarium - nature08850.pdf. Nature *464*, 367–373.

Martin, F., Aerts, A., Ahrén, D., Brun, A., Danchin, E., Duchaussoy, F., Gibon, J., Kohler, A., Lindquist, E., and Pereda, V. (2008). The genome of Laccaria bicolor provides insights into mycorrhizal symbiosis. Nature *452*, 88–92.

Martinez, D., Larrondo, L.F., Putnam, N., Gelpke, M.D.S., Huang, K., Chapman, J., Helfenbein, K.G., Ramaiya, P., Detter, J.C., and Larimer, F. (2004). Genome sequence of the lignocellulose degrading fungus Phanerochaete chrysosporium strain RP78. Nat Biotechnol *22*, 695–700.

Matsuzaki, M., Misumi, O., Shin-i, T., Maruyama, S., Takahara, M., Miyagishima, S.-Y., Mori, T., Nishida, K., Yagisawa, F., and Nishida, K. (2004). Genome sequence of the ultrasmall unicellular red alga Cyanidioschyzon merolae 10D. Nature *428*, 653–657.

Merchant, S.S., Prochnik, S.E., Vallon, O., Harris, E.H., Karpowicz, S.J., Witman, G.B., Terry, A., Salamov, A., Fritz-Laylin, L.K., Maréchal-Drouard, L., et al. (2007). The Chlamydomonas genome reveals the evolution of key animal and plant functions. Science *318*, 245–250.

Ohm, R.A., de Jong, J.F., Lugones, L.G., Aerts, A., Kothe, E., Stajich, J.E., de Vries, R.P., Record, E., Levasseur, A., and Baker, S.E. (2010). Genome sequence of the model mushroom Schizophyllum commune. Nat Biotechnol *28*, 957–963.

Palenik, B., Grimwood, J., Aerts, A., Rouzé, P., Salamov, A., Putnam, N., Dupont, C., Jorgensen, R., Derelle, E., Rombauts, S., et al. (2007). The tiny eukaryote Ostreococcus provides genomic insights into the paradox of plankton speciation. *104*, 7705–7710.

Price, D.C., Chan, C.X., Yoon, H.S., Yang, E.C., Qiu, H., Weber, A.P.M., Schwacke, R., Gross, J., Blouin, N.A., Lane, C., et al. (2012). Cyanophora paradoxa genome elucidates origin of photosynthesis in algae and plants. Science *335*, 843–847.

Prochnik, S.E., Umen, J., Nedelcu, A.M., Hallmann, A., Miller, S.M., Nishii, I., Ferris, P., Kuo, A., Mitros, T., Fritz-Laylin, L.K., et al. (2010). Genomic analysis of organismal complexity in the multicellular green alga Volvox carteri. Science *329*, 223–226.

Puiu, D., Enomoto, S., Buck, G.A., Abrahamsen, M.S., and Kissinger, J.C. (2004). CryptoDB: the Cryptosporidium genome resource. Nucleic Acids Res *32*, D329–D331.

Putnam, N.H., Butts, T., Ferrier, D.E., Furlong, R.F., Hellsten, U., Kawashima, T., Robinson-Rechavi, M., Shoguchi, E., and Yu, A.T.J.-K. (2008). The amphioxus genome and the evolution of the chordate karyotype. Nature *453*, 1064–1071.

Putnam, N.H., Srivastava, M., Hellsten, U., Dirks, B., Chapman, J., Salamov, A., Terry, A., Shapiro, H., Lindquist, E., and Kapitonov, V.V. (2007). Sea anemone genome reveals ancestral eumetazoan gene repertoire and genomic organization. Science *317*, 86–94.

Radakovits, R., Jinkerson, R.E., Fuerstenberg, S.I., Tae, H., Settlage, R.E., Boore, J.L., and Posewitz, M.C. (2012). Draft genome sequence and genetic transformation of the oleaginous alga Nannochloropis gaditana. Nature Communications *3*, 686.

Read, B.A., Kegel, J., Klute, M.J., Kuo, A., Lefebvre, S.C., Maumus, F., Mayer, C., Miller, J., Monier, A., and Salamov, A. (2013). Pan genome of the phytoplankton Emiliania underpins its global distribution. Nature.

Rensing, S.A., Lang, D., Zimmer, A.D., Terry, A., Salamov, A., Shapiro, H., Nishiyama, T., Perroud, P.-F., Lindquist, E.A., Kamisugi, Y., et al. (2008). The Physcomitrella genome reveals evolutionary insights into the conquest of land by plants. Science *319*, 64–69.

Rhind, N., Chen, Z., Yassour, M., Thompson, D.A., Haas, B.J., Habib, N., Wapinski, I., Roy, S., Lin, M.F., Heiman, D.I., et al. (2011). Comparative Functional Genomics of the Fission Yeasts. Science.

Schneider, J., Rupp, O., Trost, E., Jaenicke, S., Passoth, V., Goesmann, A., Tauch, A., and Brinkrolf, K. (2012). Genome sequence of Wickerhamomyces anomalus DSM 6766 reveals genetic basis of biotechnologically important antimicrobial activities. FEMS Yeast Res *12*, 382–386.

Sharpton, T.J., Stajich, J.E., Rounsley, S.D., Gardner, M.J., Wortman, J.R., Jordar, V.S., Maiti, R., Kodira, C.D., Neafsey, D.E., Zeng, Q., et al. (2009). Comparative genomic analyses of the human fungal pathogens Coccidioides and their relatives. Genome Res *19*, 1722–1731.

Shoguchi, E., Shinzato, C., Kawashima, T., and Gyoja, F. (2013). Draft Assembly of the Symbiodinium minutum Nuclear Genome Reveals Dinoflagellate Gene Structure. Current Biology.

Simakov, O., Marletaz, F., Cho, S.-J., Edsinger-Gonzales, E., Havlak, P., Hellsten, U., Kuo, D.-H., Larsson, T., Lv, J., Arendt, D., et al. (2013). Insights into bilaterian evolution from three spiralian genomes. Nature *493*, 526–531.

Sodergren, E., Weinstock, G.M., Davidson, E.H., Cameron, R.A., Gibbs, R.A., Angerer, R.C., Angerer, L.M., Arnone, M.I., Burgess, D.R., and Burke, R.D. (2006). The genome of the sea urchin Strongylocentrotus purpuratus. Science *314*, 941–952.

Srivastava, M., Begovic, E., Chapman, J., Putnam, N.H., Hellsten, U., Kawashima, T., Kuo, A., Mitros, T., Salamov, A., and Carpenter, M.L. (2008). The Trichoplax genome and the nature of placozoans. Nature *454*, 955–960.

Srivastava, M., Simakov, O., Chapman, J., Fahey, B., Gauthier, M.E., Mitros, T., Richards, G.S., Conaco, C., Dacre, M., and Hellsten, U. (2010). The Amphimedon queenslandica genome and the evolution of animal complexity. Nature *466*, 720–726.

Stajich, J.E., Wilke, S.K., Ahrén, D., Au, C.H., Birren, B.W., Borodovsky, M., Burns, C., Canbäck, B., Casselton, L.A., Cheng, C.K., et al. (2010). Insights into evolution of multicellular fungi from the assembled chromosomes of the mushroom Coprinopsis cinerea (Coprinus cinereus). *107*, 11889–11894.

Sucgang, R., Kuo, A., Tian, X., Salerno, W., Parikh, A., Feasley, C.L., Dalin, E., Tu, H., Huang, E., and Barry, K. (2011). Comparative genomics of the social amoebae Dictyostelium discoideum and Dictyostelium purpureum. Genome Biol *12*, R20.

Suga, H., Chen, Z., de Mendoza, A., and Sebé-Pedrós, A. (2013). The Capsaspora genome reveals a complex unicellular prehistory of animals. Nature.

Tisserant, E., Malbreil, M., and Kuo, A. (2013). Genome of an arbuscular mycorrhizal fungus provides insight into the oldest plant symbiosis.

Venter, J.C., Adams, M.D., Myers, E.W., Li, P.W., Mural, R.J., Sutton, G.G., Smith, H.O., Yandell, M., Evans, C.A., and Holt, R.A. (2001). The sequence of the human genome. Science *291*, 1304–1351.

Wang, L., Chen, W., Feng, Y., Ren, Y., Gu, Z., Chen, H., Wang, H., Thomas, M.J., Zhang, B., Berquin, I.M., et al. (2011). Genome characterization of the oleaginous fungus Mortierella alpina. PLoS ONE *6*, e28319.

Worden, A.Z., Lee, J.-H., Mock, T., Rouzé, P., Simmons, M.P., Aerts, A.L., Allen, A.E., Cuvelier, M.L., Derelle, E., and Everett, M.V. (2009). Green evolution and dynamic adaptations revealed by genomes of the marine picoeukaryotes Micromonas. Science *324*, 268–272.

(2013). A New Genetic Linkage Map of the Zygomycete Fungus Phycomyces blakesleeanus. *8*, e58931.
